# Sleep duration and treatment compliance: a population-based cross-sectional study of hypertensive patients in Bangladesh

**DOI:** 10.1186/s13104-016-2075-6

**Published:** 2016-05-13

**Authors:** Ahmed Hossain, Orin Mithila

**Affiliations:** Department of Public Health, North South University, Dhaka, Bangladesh; Apollo Hospital, Dhaka, Bangladesh

**Keywords:** Treatment compliance, Hypertension, Sleep duration, Bangladesh

## Abstract

**Background:**

Treatment with appropriate medication is a key factor to control hypertension and reduce the associated risk of complications. However, compliance with treatment is often sub-optimal, especially in developing countries. Our aim in this cross-sectional study is to investigate whether there is an association between sleep duration and treatment compliance among skilled professionals who are experiencing hypertension.

**Methods:**

A questionnaire was given to all skilled professionals who are found hypertensive in an organization of Bangladesh. To assess treatment compliance, questions on self-reported compliance test were used. We collected information on self-reported short sleep duration (6 h or less) along with socio-demographic factors and clinical conditions of the subjects.

**Results:**

Sleep duration is associated with compliance with treatment among hypertensive skilled professionals. We found overall associations of sleep duration (odds ratio (OR) 3.77, confidence interval 1.44–10.83) with treatment compliance among hypertensive patients. In addition, body mass index (OR 1.19), marital status (OR 0.16) and duration of having hypertension are found significant factors for non-compliance with treatment.

**Conclusion:**

There is an association between sleep duration and treatment compliance among the hypertensive patients. However, the study is conducted with a small group of skilled professionals from an organization and it is important to include multi-centers to validate the conclusion.

**Electronic supplementary material:**

The online version of this article (doi:10.1186/s13104-016-2075-6) contains supplementary material, which is available to authorized users.

## Background

Blood pressure control remains an essential and continuous approach to prevent the occurrence of coronary heart disease, heart failure, stroke and premature death [1]. It is also one of the most effective ways to retard the progression of diabetic and non-diabetic renal diseases [1]. Patient, non-compliance with treatment, has been recognized as one of the major reasons why antihypertensive treatments fail. A study in USA supports the statement about failing of antihypertensive treatments failure because of non-compliance [2]. In a survey, non-compliance was responsible for 17 % of treatment interruptions in Germany [3]. In Bangladesh, there were no such studies conducted till now. Therefore it is important to find the characteristic of the hypertensive populations who are non-compliant with the anti-hypertensive treatment. It will help to take necessary steps to this population to become compliant with the method of the treatment.

Non-compliance is very common among patients with a well-controlled blood pressure [4]. Thus, compliance is a very dynamic process; patients may adhere completely to their treatment for some periods of time and suddenly become noncompliant because of problems interfering with their treatments [5]. Therefore, compliance is a difficult parameter to assess clinically.

Duration of sleep plays an important role in good health and well being throughout everyone’s life. However, as a hallmark of modern society, sleep duration is decreasing for adults in the last few decades [7]. Short sleep duration is associated with decreased levels of leptin, glucose tolerance and insulin sensitivity, as well as increased levels of hunger and appetite [8]. Sleep restriction could also affect exogenous factors such as food choice and increased time available to eat. Moreover, evidence suggests the relationship between short sleep and specific behavior, such as low physical activity and low consumption of fruit and vegetables [9]. Not many studies are conducted to see the affect of sleep duration on treatment compliance among hypertensive patients. Here our interest is to examine the affects of sleep loss on treatment compliance among a group of hypertensive patients.

Bangladesh Non-communicable disease (NCD) Risk Factor Survey in 2010 reported that, approximately 20 % of adult and 40 to 65 % of elderly people suffer from hypertension in Bangladesh [10]. Poor compliance with the treatment is very common among the Bangladesh population especially among the poor people. But, it is unknown why the treatment non-compliance is present among the hypertensive patients who do have formal education (at least attended the university) and who do have jobs as skilled professionals. This group of people can afford to buy medicine and can continue to control their hypertension. These motivate us to conduct a study to investigate among the hypertensive patients to find the factors associated with treatment non-compliance.

## Methods

### The data

The study was conducted in Intertek ltd., which is located at Dhaka, Bangladesh. There were about 512 skilled professionals working in Intertek Dhaka, Bangladesh. During beginning of each year all the employees go through an employment health check up. From the history of the employee profiles we found 123 employees who were diagnosed as hypertensive patients for at least 6 months and they were prescribed for medication to control hypertension. These employees are educated who had a university education before joining in Intertek. We conducted a population based cross-sectional survey among these skilled professionals and collected data for 101 employees. Other 22 employees either declined to fill the questionnaire or they were on vacation during the survey. The Additional file 1 gives the data in excel format which can be downloaded from http://individual.utoronto.ca/ahmed_3/index_files/data/data.html.

### Compliance with treatment

Wong et al. [11] delineated the treatment compliance when medical or health advice coincides with the individual’s behavior with regard to the use of medication, recommended changes in lifestyle, and attendance to medical appointments. The Morisky et al. [6] portrayed the treatment compliance in terms of four items coded as “yes” or “no”. These items relate to not taking medications due to carelessness, forgetting, feeling better, or feeling worse. Morisky et al. [6] gave emphasis on forgetting medication use to define the treatment compliance which can be improved by a reminder from family members. We measured the compliance by asking three questions in a self-reported compliance test (SC) following suggestions from the Morisky-Green test (MG) and Wong et al. [11]. The three questions include: (1) whether the patient continue with the medicine [6, 11], (2) whether the patient continue with regular clinic attendance [11], and (3) whether the patient get social support from family members or friends who were concerned about the respondents hypertension or who were helpful in reminding the respondent about taking medication [6, 11]. The first question about compliance with medication is categorized as: “compliance: yes” (where the respondent ‘never misses’ or ‘rarely misses’ to take his/her medication doses); and “compliance: no” (where the respondent ‘regularly’ or ‘fairly regularly’ misses to take his/her medication). All the questions are given a score of 1. Among these three questions we defined the compliance with treatment as “yes” if an individual is found with total score of two including a positive response of the first question.

### Independent variables

A self-reported questionnaire was designed to obtain information on gender, date of birth, height, weight, date of survey, marital status (married or unmarried), other health related problems (yes or no), family history of hypertension (yes or no), duration of hypertension, smoking habit (yes or no), duration of high blood pressure and average daily sleep duration. Age was calculated in years from the date of births and body mass index (BMI) was calculated from the height and weight. The age and BMI were treated as continuous variables. The duration of hypertension was categorized as ≤1 years, 2–3 years and ≥4 years. Sleep duration was assessed by the question, “How many hours each day do you spend sleeping?”. Sleep duration was categorized as short sleep duration (<6 h) and normal sleep duration (≥6 h). From August to September 2015, inspectors who were familiar with the questionnaire went to Intertek and distributed the questionnaire among the skilled professionals who were known to have hypertension for at least 6 months and taking medication to control it. Any question or confusion from the participants was clarified to ensure that everyone understood all of the items. The completed questionnaires were checked for quality control.

### Ethical approval

Ethical approval for the study protocol was obtained from the North South University Review Committee and Intertek, Dhaka and a written informed consent was obtained from all the participants.

### Statistical analysis

We analyzed the data using software R. Descriptive statistics were calculated for all of the variables, including continuous variables (presented as boxplots) and categorical variables (presented as frequencies). The unadjusted association between each of categorical variables and treatment compliance was evaluated by Chi square test. The association between sleep duration and treatment compliance was estimated by multivariable logistic regression models, which were adjusted for age, gender, marital status, BMI, other health problem, family history of hypertension, smoking status and duration of high blood pressure into the model. The results were reported by odds ratios (ORs) and corresponded 95 % confidence intervals (CIs). P-values less than 0.05 were considered statistically significant.

## Results

**Fig. 1:**
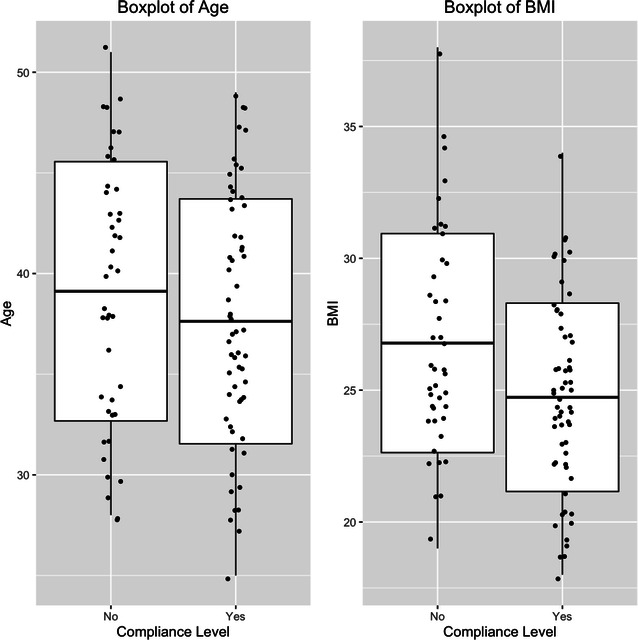
*Boxplot* of Age and BMI corresponding to compliance levels

The data comprised 101 skilled workers aged 25–51 years (mean 38.27 years, 72.27 % male). The baseline characteristics of the participants, such as sex, marital status, family history of hypertension, other health problems, current smoking status, sleep duration and duration of blood pressure, are described (see Table 1). Compared with a female, a male had a higher prevalence of non-compliant, but the *p* value does not indicate sex has any significant effect on compliance (p > 0.05, Table 1). No variable is found significant on compliance with treatment considering 5 % significance level. The age and BMI are considered as a continuous variable and the boxplots for age and BMI corresponding to compliance level are given in the Fig. 1. It appears from the Fig. 1 (boxplot for age) that younger individuals are more compliant about treatment to control hypertension compared to the older people. Again, the boxplot for BMI indicates that weighted individuals are more non-compliant with treatment compared to those who have less BMI. The figure shows that the most persons who are having BMI greater than 30 are in the non-compliant group.

**Table 1:** Participant characteristic by treatment compliance and unadjusted association between each of covariates and treatment compliance

Variables	Categories	Compliance level		χ^2^-value	p value
		No	Yes		
Sex	F	14	14	0.701	0.402
	M	28	45		
Marital status	Married	38	45	2.48	0.115
	Unmarried	4	14		
Other health problems	No	16	27	0.318	0.572
	Yes	26	32		
Family history	Negative	13	14	0.336	0.561
	Positive	29	45		
Smoking	No	30	43	0	1
	Yes	12	16		
Sleep hour	6 h	28	28	2.928	0.087
	<6 h	14	31		
Use of sleep drug	No	28	41	0.007	0.933
	Yes	14	18		
Duration of hypertension	1 year	7	7		
	2–3 years	17	23	0.631	0.729
	4 years	18	29

Moreover, sleep duration is related to obesity [12, 13]. Therefore, it is important to stratify obesity to avoid the confounding effect in the analysis. We found the correlation between age and BMI is 0.14, which indicates a weak relationship between age and BMI, and therefore BMI is not considered as a confounding variable in this study. Here we considered BMI as a continuous variable and continued our multivariate analysis without stratification by obesity.

We fit a multivariable logistic regression model with treatment compliance levels after adjusting all the potential risk factors. The adjusted odds ratios (ORs) for compliant group are given in Table 2. It appears that the 95 % confidence interval of sleep duration (OR = 3.776, CI = 1.445, 10.834) is not including the value 1 and therefore it is a significant variable. In fact, the p-value is found 0.009, which indicates a significant relationship between, sleep duration and treatment compliance at 5 % significance level. Again, the odds ratio explains that the odds of being non-compliant are 3.78 times more for the long sleep duration group compared to the group who sleep less than 6 h. It is expected because many patients may feel well of having a long sleep and they may have a negative attitude towards taking medication. In addition, the marital status (OR = 0.157, CI = 0.024, 0.789; p-value = 0.034) and BMI (OR = 1.19, CI = 1.05, 1.3; p-value = 0.008) are found significant. The married professionals are about 80 % less likely to be treatment compliant compared to the unmarried professionals. Again, one unit increase of BMI indicates 18 % more likely to be treatment compliant to control hypertension.

**Table 2:** Adjusted relationships between covariates and treatment compliance that is analyzed using logistic regression

Variable	Reference	Estimate	OR	LCL	UCL	p value
Age		0.036	1.036	0.936	1.151	0.495
Sex- female	Male	−0.383	0.682	0.225	2.054	0.494
Marital status- married	Unmarried	−1.851	0.157	0.024	0.789	0.034
BMI		0.173	1.189	1.052	1.364	0.008
Other problem	Yes	−0.38	0.684	0.209	2.168	0.521
Family history	Positive	−0.538	0.584	0.2	1.657	0.313
Smoking- no	Yes	0.854	2.348	0.79	7.285	0.129
Sleep hour- short	Long (>6 h)	1.329	3.776	1.445	10.834	0.009
Duration of hypertension- 2–3 Y	1 Y	−1.656	0.191	0.028	1.052	0.069
Duration of Hypertension- 4 Y	1 Y	−1.743	0.175	0.027	0.916	0.049

*OR* odds ratio

*LCL* 95 % Lower confidence limit of OR

*UCL* 95 % Upper confidence limit of OR

## Discussions and conclusion

Treatment with appropriate medication and compliance with the treatment are key factors in the control of hypertension. The failure to control hypertension takes an undesirable toll on patients and their families. Non-compliance with treatment has a cost as it leads to an increase in medical expenses and it decreases the cost effectiveness of the interventions. Therefore, it is important to identify the hypertensive patients who are at risk of non-compliance with treatment. In this study we found, patients with long sleep duration are more non-compliant with treatment among the skilled hypertensive professionals. In addition, unmarried patients are more non-compliant with the treatment of hypertension. Obese patient tend to be more treatment non-compliant than the normal weight patients. Therefore, it is important to give attention to overweight and obese patients so that they can be treatment compliant to control hypertension. Consideration should also be given to long-term hypertensive patients. There is a tendency among the long-term hypertensive patients of poor adherence to the treatment and want to avoid clinician’s advice. In conclusion, our attention should be focused on unmarried, obese patients who have a habit of long sleep duration so that they can take a suitable treatment plan to control hypertension. The study involves a relatively small number of participants, which means additional studies need to be conducted in order to define more precisely the hypertensive populations which are the most likely to benefit from monitoring.

## Additional file

The data file is available from http://individual.utoronto.ca/ahmed_3/index_files/data/data.html.

10.1186/s13104-016-2075-6 The data is in excel format.
